# Association study of *AFF1* rs340630 polymorphism with genetic susceptibility to rheumatoid arthritis in Chinese population

**DOI:** 10.1590/1414-431X20187126

**Published:** 2018-05-17

**Authors:** Qing-Qing Sun, Dong-Jin Hua, Si-Chao Huang, Han Cen, Li Zhou, Song Shao

**Affiliations:** 1 Department of Preventive Medicine, Medical School of Ningbo University, Ningbo, Zhejiang, China; 2 Zhejiang Provincial Key Laboratory of Pathophysiology, School of Medicine, Ningbo University, Ningbo, Zhejiang, China; 3 Department of Rheumatology, Ningbo First Hospital, Ningbo Hospital of Zhejiang University, Ningbo, Zhejiang, China; 4 Department of Orthopaedics, Liu'an People's Hospital, Liu'an, Anhui, China

**Keywords:** Rheumatoid arthritis, AFF1, Polymorphism, Chinese

## Abstract

This study was performed to examine whether the *AF4/FMR2 family, member 1* (*AFF1*) rs340630 polymorphism is involved in the genetic background of rheumatoid arthritis (RA) in a Chinese population. Two different study groups of RA patients and controls (328 RA patients and 449 healthy controls in the first study group; 232 RA patients and 313 controls in the second study group) were included in our study. Overall, there was no significant difference in either genotype (P=0.71 and 0.64 in the first and second study group, respectively) nor allele (in the first study group: A *vs* G, P=0.65, OR=1.05, 95%CI=0.85–1.29; in the second study group: G *vs* A, P=0.47, OR=1.10, 95%CI=0.86–1.40) frequencies of *AFF1* rs340630 polymorphism between RA patients and controls. Our study represents the first report assessing the association of *AFF1* rs340630 polymorphism with RA risk. No significant evidence was found for the dominant or recessive models. Further case-control studies with larger sample sizes and fine-mapping studies are needed to clarify the role of *AFF1* in the genetic basis of RA.

## Introduction

Rheumatoid arthritis (RA) is a chronic autoimmune disease characterized by persistent synovitis, systemic inflammation, and the presence of autoantibodies. If the optimal therapeutic opportunity is missed, cartilage and bone damage as well as disability would ensue (1). As a multifactorial disease, both genetic and environmental factors have been implicated in the onset and progression of RA, and the heritability of RA has been estimated to fall into the range of 40∼60% (2). During the past decade, due to the success of genome-wide association studies (GWASs) and large-scale candidate gene studies, a large number of loci/genes susceptible to RA have been discovered and confirmed. However, all these known genetic risk factors could only account for a small fraction of the heritability of RA (3), indicating that a variety of susceptible genes associated with RA have to be discovered.

Systemic lupus erythematosus (SLE) is another prototypical autoimmune disease, characterized by the production of a wide range of autoantibodies against self-components of the cell nucleus and multiple organ involvement (4). Several similar clinical manifestations such as arthritis and auto-antibody production have been noted between RA and SLE, and their co-existence and familial aggregation have been documented in epidemiological studies (5–7). This suggests that genetic risk factors might be shared between RA and SLE, as seen in the recent meta-analyses and investigations in which susceptible genes associated with one are replicated in the other (8). To date, many susceptible genes, such as *HLA-DRB1*, *PTPN22*, *STAT4*, *TNFAIP3*, *FCGR2A,* and *IRF5* have been detected to be shared between RA and SLE (9,10). Thus, the strategy of replicating associations of RA with polymorphisms significantly associated with SLE might provide new insights for identifying polymorphisms involved in the genetic background of RA.

Recently, GWAS and multi-staged replication studies integrating quantitative trait loci expression performed in Japanese populations have identified a single-nucleotide polymorphism (SNP), rs340630, within the *AF4/FMR2 family, member 1* (*AFF1*) associated with SLE at genome-wide significance level (11). Notably, another polymorphism, rs10865035, located in the promoter region of *AF4/FMR2 family, member 3* (*AFF3*), which also belongs to the AFF gene family, was found to be significantly associated with RA (12). Subsequently, the genetic association of *AFF3* with RA has been replicated in several studies (13–15) and RA GWAS meta-analyses (16,17). Because of the shared genetic background of SLE and RA, the association between *AFF*3 rs10865035 polymorphism and SLE genetic predisposition has been replicated in a Chinese population (18). It is this successful replication and because *AFF*1 and *AFF*3 belong to the same gene family that *AFF*1 makes a good candidate for RA. However, to the best of our knowledge no study has been reported regarding this relationship. Thus, the aim of our study was to assess whether the *AFF*1 rs340630 polymorphism is implicated in the genetic background of RA in Chinese populations.

## Material and Methods

### Patients and controls

Two different study groups of RA patients and healthy controls were included in the present study.

The first study group consisted of 328 RA patients (51 males and 277 females, mean±SD age 53.77±11.99 years) and 449 healthy control subjects (281 males and 168 females, mean±SD age 51.10±15.47 years). Patients with RA were in- or outpatients enrolled in the Department of Rheumatology, Ningbo First Hospital. The healthy controls were from the physical examination center of the same hospital, and individuals with any signs or symptoms of autoimmune diseases were excluded. All patients were diagnosed according to the American College of Rheumatology 1987 revised criteria for the classification of RA (19) or the 2010 RA classification criteria (20). This study was reviewed and approved by the Ethics Committee of Ningbo University, and informed consent was obtained from all participants.

The second study group included 232 unrelated patients having RA (187 females and 45 males, mean±SD age 48.69±14.18 years), who were consecutively recruited from Liu'an People's Hospital. All patients met the American College of Rheumatology 1987 revised criteria for the classification of RA (19). The control group was comprised of 313 ethnically-matched patients (116 females and 197 males, mean±SD age 47.02±16.34 years) admitted to the same hospital due to the treatment of trauma, and all of them were without any symptoms or signs of autoimmune diseases. This study was reviewed and approved by the Ethics Committee of Liu'an People's hospital, and informed consent was obtained from all subjects.

All procedures performed in studies involving human participants were in accordance with the ethical standards of the institutional and/or national research committee, and with the 1964 Helsinki Declaration and its later amendments or comparable ethical standards.

### DNA extraction and SNP genotyping

EDTA anti-coagulated venous blood samples were collected from all participants. In the first case-control study, genomic DNA was extracted from peripheral blood lymphocytes using an automatic nucleotide acid extraction instrument in accordance with the standard procedures of the corresponding DNA extraction kit (Tianlong, China). In the second case-control study, genomic DNA was extracted from peripheral blood lymphocytes according to the standard procedures of the Flexi Gene¯ DNA Kit (Qiagen, USA). The genotype of *AFF1* rs340630 polymorphism in the first study group was determined by Shanghai Biowing Applied Biotechnology Co. Ltd. (www.biowing.com.cn), applying the ligase detection reaction-polymerase chain reaction (LDR-PCR) technology, and the same polymorphism of participants in the second study group was detected by Shanghai Genesky Bio-Tech Co, Ltd (www.geneskybiotech.com) using the SNaPshot Assay.

### Statistical analysis

Hardy-Weinberg equilibrium (HWE) of the *AFF1* rs340630 polymorphism genotype distribution among the control group was assessed using the chi-square goodness of fit test. The chi-square test was used to compare the allele frequencies between RA patients and controls. The difference in genotype frequencies between them and the associations of RA with the risk allele of the *AFF1* rs340630 polymorphism under different genetic models (dominant and recessive models) were evaluated by logistic regression with the adjustment of gender and/or age. All corresponding odds ratios (ORs) and 95% confidence intervals (95%CI) were calculated, and a statistically significant difference was determined if a two-tailed P value was <0.05. All statistical analyses were performed by PASW Statistics 18.0 software (SPSS, Inc., USA).

## Results

The genotype and allele frequencies of the *AFF1* rs340630 polymorphism among two study groups are shown in Table 1 and Table 2, respectively. The P values for the HWE test of the *AFF1* rs340630 polymorphism genotype distribution among the control group were 0.04 and 0.93 in the first and the second study group, respectively.

**Table 1.** Genotype and allele frequencies distribution of *AFF1* rs340630 polymorphism in the first cohort of RA patients and controls.

*AFF1* rs340630	RA patients (n=327)	Controls (n=446)	P value	OR (95%CI)
Genotype				
A/A	116 (35.5)	157 (35.2)	0.71	
A/G	164 (50.2)	216 (48.4)		
G/G	47 (14.4)	73 (16.4)		
Allele				
A	396 (60.6)	530 (59.4)	0.65	1.05 (0.85–1.29)
G	258 (39.4)	362 (40.6)		
Dominant model				
A/A+A/G	280 (85.6)	373 (83.6)	0.50	1.17 (0.74–1.86)
G/G	47 (14.4)	73 (16.4)		
Recessive model				
A/A	116 (35.5)	157 (35.2)	0.50	1.13 (0.80–1.59)
A/G+G/G	211 (64.5)	289 (64.8)		

Data are reported as n (%). RA: rheumatoid arthritis; OR: odds ratio; CI: confidence interval. Regarding genotype comparison and associations under the genetic models (dominant model and recessive model), the P values and corresponding ORs (95%CI) were calculated with adjustment for age and gender.

**Table 2.** Genotype and allele frequencies distribution of *AFF1* rs340630 polymorphism in the second cohort of RA patients and controls.

*AFF1* rs340630	RA patients (n =232)	Controls (n=313)	P value	OR (95%CI)
Genotype				
G/G	42 (18.1)	42 (13.4)	0.64	
A/G	113 (48.7)	168 (53.7)		
A/A	77 (33.2)	103 (32.9)		
Allele				
G	197 (42.5)	252 (40.3)	0.47	1.10 (0.86–1.40)
A	267 (57.5)	374 (59.7)		
Dominant model				
G/G+A/G	155 (66.8)	210 (67.1)	0.48	1.16 (0.77–1.73)
A/A	77 (33.2)	103 (32.9)		
Recessive model				
G/G	42 (18.1)	42 (13.4)	0.42	1.24 (0.74–2.07)
A/G+A/A	190 (81.9)	271 (86.6)		

Data are reported as n (%). RA: rheumatoid arthritis; OR: odds ratio; CI: confidence interval. Regarding genotype comparison and associations under the genetic models (dominant model and recessive model), the P values and corresponding ORs (95%CI) were calculated with adjustment for gender.

### Association of *AFF1* rs340630 polymorphism with the risk of RA in the first study group

In the first study group, there was a significant difference in age distribution (P=7.07×10^−3^) and gender composition (P<0.01) between RA patients and healthy controls. Thus, the variation in genotype distribution between RA cases and controls, and the associations between RA and risk allele of the *AFF1* rs340630 polymorphism under dominant and recessive models were evaluated by logistic regression, with the adjustment of age and gender. As shown in Table 1, no significant difference was found in either the genotype distribution of the *AFF1* rs340630 polymorphism between RA patients and healthy controls (P=0.71), or in the allele distribution (A *vs* G, P=0.65, OR=1.05, 95%CI=0.85–1.29). We also evaluated the associations between the major allele A of *AFF1* rs340630 polymorphism and the risk of RA under the dominant and recessive model. However, no significant evidence was found (A/A+A/G *vs* G/G, P=0.50, OR=1.17, 95%CI=0.74–1.86; A/A *vs* A/G+G/G, P=0.50, OR=1.13, 95%CI=0.80–1.59).

### Association of *AFF1* rs340630 polymorphism with the risk of RA in the second study group

In the second study group, we found a significant difference in gender composition (P*<*0.01) between RA patients and controls, and a non-significant difference in age distribution (P=0.20). Thus, the variance in genotype distribution between RA cases and controls, and the associations of RA with the risk allele of the *AFF1* rs340630 polymorphism under the dominant and recessive model were evaluated by logistic regression with the adjustment of gender. As shown in Table 2, no significant difference was found (P*=*0.64). The minor allele G was increased in patients with RA compared to controls, but this change did not reach statistically significant levels (G *vs* A, P=0.47, OR=1.10, 95%CI=0.86–1.40). The associations of the minor allele G of the *AFF1* rs340630 polymorphism with the risk of RA under the dominant and recessive model were also assessed, but again no significance was detected (G/G+A/G *vs* A/A, P=0.48, OR=1.16, 95%CI=0.77–1.73; G/G *vs* A/G+A/A, P=0.42, OR=1.24, 95%CI=0.74–2.07).

## Discussion

It has been increasingly recognized that multiple genes might be shared by distinct autoimmune diseases (8–10), including the genetic background of RA and SLE. In the present study, we have adopted the strategy of replicating the association of RA with genetic polymorphism within *AFF1*, which was previously found to be associated with SLE in a GWAS and in multi-staged replication studies integrating the expression of quantitative trait loci in a Japanese population (11). However, our results revealed that the polymorphism was not associated with RA in a Chinese population. This might be because *AFF1* is not really involved in the genetic basis of RA, though it should be noted that our study's relatively low capability of detecting mild associations may have affected the results.

The AFF gene family consists of *AFF1* [also known as ALL1-fused gene from chromosome 4 (*AF4*)], *AFF2* [also known as fragile X mental retardation 2 (*FMR2*)], *AFF3* [also known as lymphoid nuclear protein related to AF4 (*LAF4*)], and *AFF4* [also known as ALL1-fused gene from 5q31 (*AF5q31*) or major CDK9 elongation factor associated-protein (*MCEF*)] (21). By targeting polymorphisms showing suggestive evidence for association with type 1 diabetes (T1D), Barton et al. (12) identified a novel genetic polymorphism in *AFF3* for RA. Subsequently, the association between the genetic predisposition of *AFF3* and RA has been validated in different investigations (13–15), including GWASs meta-analyses of RA performed in Chinese and Europeans (16,17). Of note, in view of the potential genetic basis shared between RA and SLE, the association of *AFF3* with genetic susceptibility to SLE was replicated in a study conducted in a Chinese population (18).

In a GWAS and in multi-staged replication studies carried out in a Japanese population, a new genetic association between *AFF1* rs340630 polymorphism and genetic predisposition to SLE with genome-wide significance level was identified (11). Additionally, the risk allele of *AFF1* rs340630 polymorphism was found to be associated with the increase in transcript levels of *AFF1*, indicating this polymorphism is functional (11). *AFF1* was first identified as a fusion partner with myeloid/lymphoid, or mixed-lineage leukemia (MLL) in an acute lymphoblastic leukemia (ALL) patient (22), being predominantly expressed in a lymphocyte system (23,24). The essential role of *AFF1* in the development of lymphocytes was originally elucidated by *AFF1*-null mice (25). In the report by Okada et al. (11), the tissue distribution of AFF1 has also been investigated, and their results revealed that *AFF1* transcripts were prominently expressed in CD4^+^ and CD19^+^ peripheral blood lymphocytes. Taken together, these observations indicate that *AFF1* is implicated in the development of lymphocytes, and its altered expression, caused by allele substitution, might contribute to the development of autoimmune diseases (26). Based on the above-mentioned background, our study was inspired by these facts: the shared genetic architecture of RA and SLE (8–10); *AFF1* and *AFF3* belonging to the same genetic family (21); the success of replicating the association of *AFF3* with SLE by targeting polymorphism related to RA (18); and the potential biological function of *AFF1* in lymphocyte development (11,23–25). Nevertheless, we did not find any significant association signal in our two different study groups.

Notably, replication studies conducted in Chinese (18) and Iranian (27) populations did not find significant evidence for the association of *AFF1* rs340630 polymorphism with SLE. This might be due to the smaller sample sizes or the different linkage disequilibrium patterns across different populations. Thus, further investigation is needed.

In conclusion, our study represents the first report examining the association of *AFF1* rs340630 polymorphism with genetic susceptibility to RA in a Chinese population, and no significant evidence was found. Further independent case-control studies with lager sample sizes in different ethnic populations and fine-mapping studies are needed to clarify the role of *AFF1* in the genetic basis of RA.

## References

[1] Smolen JS, Aletaha D, McInnes IB (2016). Rheumatoid arthritis. Lancet.

[2] Terao C, Raychaudhuri S, Gregersen PK (2016). Recent advances in defining the genetic basis of rheumatoid
arthritis. Annu Rev Genomics Hum Genet.

[3] Okada Y, Wu D, Trynka G, Raj C, Ikari Y, Kochi Y (2014). Genetics of rheumatoid arthritis contributes to biology and drug
discovery. Nature.

[4] Cen H, Leng RX, Wang W, Zhou M, Feng CC, Zhu Y (2013). Association study of IFIH1 rs1990760 polymorphism with systemic
lupus erythematosus in a Chinese population. Inflammation.

[5] Alarcón-Segovia D, Alarcón-Riquelme ME, Cardiel MH, Caeiro F, Massardo L, Villa AR (2005). Familial aggregation of systemic lupus erythematosus, rheumatoid
arthritis, and other autoimmune diseases in 1,177 lupus patients from the
GLADEL cohort. Arthritis Rheum.

[B06] Hemminki K, Li X, Sundquist J, Sundquist K (2009). Familial associations of rheumatoid arthritis with autoimmune
diseases and related conditions. Arthritis Rheum.

[7] Kuo CF, Grainge MJ, Valdes AM, See LC, Luo SF, Yu KH (2015). Familial aggregation of systemic lupus erythematosus and
coaggregation of autoimmune diseases in affected families. JAMA Intern Med.

[8] Cen H, Wang W, Leng RX, Wang TY, Pan HF, Fan YG (2013). Association of IFIH1 rs1990760 polymorphism with susceptibility
to autoimmune diseases: a meta-analysis. Autoimmunity.

[9] Gregersen PK, Olsson LM (2009). Recent advances in the genetics of autoimmune
disease. Annu Rev Immunol.

[10] Orozco G, Eyre S, Hinks A, Bowes J, Morgan AW, Wilson AG (2011). Study of the common genetic background for rheumatoid arthritis
and systemic lupus erythematosus. Ann Rheum Dis.

[11] Okada Y, Shimane K, Kochi Y, Tahira T, Suzuki A, Higasa K (2012). A genome-wide association study identified AFF1 as a
susceptibility locus for systemic lupus eyrthematosus in
Japanese. PLoS Genet.

[12] Barton A, Eyre S, Ke X, Hinks A, Bowes J, Flynn E (2009). Identification of AF4/FMR2 family, member 3 (AFF3) as a novel
rheumatoid arthritis susceptibility locus and confirmation of two further
pan-autoimmune susceptibility genes. Hum Mol Genet.

[13] Freudenberg J, Lee HS, Han BG, Shin HD, Kang YM, Sung YK (2011). Genome-wide association study of rheumatoid arthritis in Koreans:
population-specific loci as well as overlap with European susceptibility
loci. Arthritis Rheum.

[B14] Plant D, Flynn E, Mbarek H, Dieudé P, Cornelis F, Arlestig L (2010). Investigation of potential non-HLA rheumatoid arthritis
susceptibility loci in a European cohort increases the evidence for nine
markers. Ann Rheum Dis.

[15] Prasad P, Kumar A, Gupta R, Juyal RC, Thelma BK (2012). Caucasian and Asian specific rheumatoid arthritis risk loci
reveal limited replication and apparent allelic heterogeneity in north
Indians. PLoS One.

[16] Stahl EA, Raychaudhuri S, Remmers EF, Xie G, Eyre S, Thomson BP (2010). Genome-wide association study meta-analysis identifies seven new
rheumatoid arthritis risk loci. Nat Genet.

[17] Jiang L, Yin J, Ye L, Yang J, Hemani G, Liu AJ (2014). Novel risk loci for rheumatoid arthritis in Han Chinese and
congruence with risk variants in Europeans. Arthritis Rheumatol.

[18] Cen H, Leng RX, Wang W, Zhou M, Feng CC, Chen GM (2012). Association of AFF1 rs340630 and AFF3 rs10865035 polymorphisms
with systemic lupus erythematosus in a Chinese population. Immunogenetics.

[19] Arnett FC, Edworthy SM, Bloch DA, McShane DJ, Fries JF, Cooper NS (1988). The American Rheumatism Association 1987 revised criteria for the
classification of rheumatoid arthritis. Arthritis Rheum.

[20] Aletaha D, Neogi T, Silman AJ, Funovits J, Felson DT, Bingham CO (2010). 2010 Rheumatoid arthritis classification criteria: an American
College of Rheumatology/European League Against Rheumatism collaborative
initiative. Arthritis Rheum.

[21] Melko M, Douguet D, Bensaid M, Zongaro S, Verheggen C, Gecz J (2011). Functional characterization of the AFF (AF4/FMR2) family of
RNA-binding proteins: insights into the molecular pathology of FRAXE
intellectual disability. Hum Mol Genet.

[22] Gu Y, Nakamura T, Alder H, Prasad R, Canaani O, Cimino G (1992). The t(4;11) chromosome translocation of human acute leukemias
fuses the ALL-1 gene, related to Drosophila trithorax, to the AF-4
gene. Cell.

[23] Baskaran K, Erfurth F, Taborn G, Copeland NG, Gilbert DJ, Jenkins NA (1997). Cloning and developmental expression of the murine homolog of the
acute leukemia proto-oncogene AF4. Oncogene.

[24] Isnard P, Depetris D, Mattei MG, Ferrier P, Djabali M (1998). cDNA cloning, expression and chromosomal localization of the
murine AF-4 gene involved in human leukemia. Mamm Genome.

[25] Isnard P, Coré N, Naquet P, Djabali M (2000). Altered lymphoid development in mice deficient for the mAF4
proto-oncogene. Blood.

[26] Falgarone G, Semerano L, Rullé S, Boissier MC (2009). Targeting lymphocyte activation to treat rheumatoid
arthritis. Joint Bone Spine.

[27] Arabi E, Garshasbi M, Jamshidi AR, Khalesi R, Ahmadzadeh N, Akbarian M (2016). Association Study of an AFF1 gene polymorphism (rs340630) with
Iranian systemic lupus erythematosus patients. Acta Reumatol Port.

